# Tissue Is the Issue: A Systematic Review of Methods for the Determination of Infarct Volume in Acute Ischaemic Stroke

**DOI:** 10.3390/brainsci15060583

**Published:** 2025-05-28

**Authors:** Fatimah Al Ahmed, Patrick Kennelly, Darragh Herlihy, Jorin Bejleri, David J. Williams, John J. Thornton, Shona Pfeiffer

**Affiliations:** 1 School of Medicine, RCSI University of Medicine and Health Sciences, D02 YN77 Dublin, Ireland; fatimah.alahmed@nhs.net (F.A.A.); patrickkennelly@rcsi.com (P.K.); 2 Department of Neuroradiology, Beaumont Hospital, D09 V2N0 Dublin, Ireland; darraghherlihy@beaumont.ie (D.H.); johnthornton@beaumont.ie (J.J.T.); 3 Department Geriatric & Stroke Medicine, Beaumont Hospital, RCSI University of Medicine and Health Sciences, D09 YD60 Dublin, Ireland; jorinbejleri@gmail.com (J.B.); davidwilliams@rcsi.com (D.J.W.); 4 Department of Physiology and Medical Physics, RCSI University of Medicine and Health Sciences, D02 YN77 Dublin, Ireland

**Keywords:** ischaemic stroke, infarct measurement, infarct volume, non-contrast computerised tomography, stroke

## Abstract

Background and aims: Recent advances in acute stroke interventions have highlighted the importance of accurate determination of infarct volume in the evaluation of acute stroke patients, carrying important prognostic and therapeutic implications for treatment planning, outcome prediction, and evaluation of the success of therapeutic interventions. However, there is no consensus on the methodologies employed to measure cerebral infarct volume. We aimed to assess the reproducibility and reliability of methods employed in the clinical determination of infarct volume in acute ischaemic stroke. Methods: We carried out a systematic review of studies assessing methodologies for the determination of infarct volume in the acute phase (<24 h). We searched Medline PubMed, Scopus, Cinahl, Cochrane Library, Web of Science, and Embase for studies examining image-based diagnosis of acute ischaemic stroke < 24 h by CT or MRI. Data on patient cohorts, imaging type, time from symptoms onset, methodologies and quantification strategies, rater reliability, accuracy, sensitivity, and specificity were compared. Results: We identified eighteen eligible studies with a total of 3298 ischaemic stroke patients assessing a variety of manual, semi-automated, and fully-automated methods. The ABC/2 method was found to be highly reliable, reproducible, and accurate, and provides the best manual estimate of infarction, but has a tendency to under- or overestimate infarct volume. Semi-automated and automated approaches with user refinement showed excellent inter-rater and intra-rater correlation. However, differences in operating algorithms and lack of standardisation of image acquisition parameters, quality, and format may impact performance and reproducibility. Conclusions: Of all methods, automated and semi-automated approaches utilising rater judgment and refinement represent the most robust approaches, with semi-automated tools demonstrating consistent and repeatable results. We recommend a standardised reporting of study methodologies for the accurate interpretation and comparison of efficacy of therapeutic interventions and patient outcomes, especially in a multi-centre setting. This may allow for more effective evaluation of stroke therapies and accelerate ischaemic stroke treatment decisions.

## 1. Introduction

Stroke is one of the leading causes of death and disability (measured in disability-adjusted life years, DALYs), globally [1,2]. Ischaemic stroke, caused by a thrombotic or embolic event interrupting blood flow to the brain resulting in an area of necrotic tissue (cerebral infarct), is the most common type of stroke, accounting for ~85% of all strokes. Haemorrhagic stroke, due to bleeding into the brain by the rupture of a blood vessel, accounts for 10–15% of all strokes. Without proper blood supply, parts of the brain are deprived of oxygen and start to die, and the devastating effects of stroke often lead to poor recovery. Approximately 25% of ischaemic stroke patients can be treated with thrombolysis, available at hospitals with diagnostic imaging facilities (computerized tomography (CT)) and clinical expertise to treat stroke. Approximately 30–40% of ischaemic strokes are due to large vessel occlusion (LVO) and are potentially eligible for Endovascular Thrombectomy (EVT), a highly efficacious ground-breaking treatment for ischaemic stroke due to LVO removing the clot using stent retriever devices and/or aspiration [3].

Pathophysiological analysis after focal brain ischaemia reveals a core area of critical ischaemia (infarction) and perhaps an ischaemic penumbra, an area of under-perfused, functionally impaired but potentially viable tissue, with progressive neuronal injury and loss of function damage increasing in severity and permanence with time from onset [4,5]. Timely reperfusion of the penumbra is therefore the most effective treatment, reducing final infarct core and preserving viable ischaemic tissue, thereby improving outcomes. Recent rapid advances in acute stroke interventions, in particular the introduction of endovascular treatment approaches, have demonstrated unequivocal benefits for reperfusion of the ischaemic brain [3]. Furthermore, this area of viable tissue represents a window for therapeutic intervention for neuroprotection, to limit the progressive damage in the compromised penumbra and any potentially detrimental effects of reperfusion contributing to ischaemic injury [6,7]. Without such interventions, cells in the penumbra will also die and the core will expand [7,8,9]. Accurate evaluation of cerebral physiological status to determine eligibility for treatments requires clinical expertise and rapid access to appropriate neuroimaging diagnostic facilities, and the success of acute therapeutic interventions is time-dependent (“Time is Brain”). Importantly, the speed at which imaging is performed and the interpretation accuracy have significant consequences on patient outcomes, impacting the consideration of interventions such as thrombolysis and EVT.

Determination of infarct volume is integral to a patient-centred care approach to treatment. Infarct volume has been shown to be a reliable prognostic clinical tool in the prediction of clinical severity and functional outcome, and can be used in the identification of patients likely to suffer post-thrombolysis haemorrhage or malignant oedema, informing choice of appropriate therapeutic intervention [10,11,12,13]. Furthermore, infarct volume measurement plays an important role in investigating the efficacy of neuroprotective trials [14]. The accurate and reproducible determination of cerebral infarct volume, therefore, has important prognostic and therapeutic implications for treatment planning, outcome prediction, and evaluation of the success of therapeutic interventions.

Infarct volume is primarily measured from computed tomography (CT) or magnetic resonance imaging (MRI). Non-contrast CT (NCCT) remains the modality of choice in the acute setting due to its ready availability, speed, cost, limited exclusion criteria, and ability to rule out intracranial haemorrhage. However, due to high variability in the prevalence of early ischaemic signs on NCCT, MRI with diffusion-weighted imaging (DWI) is considered the gold standard for early stroke detection. Additional diagnostic imaging techniques, such as CT angiography (CTA), perfusion CT, and perfusion-weighted imaging (PWI), can further improve detection of infarcts, although these modalities are impacted by cost, access, expertise, and time. To assist in clinical decision-making from radiological findings, the application of the Alberta Stroke Program Early CT Score (ASPECTS) scoring system is a quantitative score that measures the extent of early ischaemic changes on brain CT and, while not perfect, is a widely validated, powerful prognostic indicator of functional outcome [4,7,10,11,12,13,14,15]. Similarly, DWI-ASPECTS has been shown to demonstrate high prognostic power, offset by increased costs and imaging time.

Currently there is no consensus on a standardised, reliable and reproducible methodology for the determination of infarct volume in the clinical setting, and approaches vary considerably across a variety of manual estimation, automated, and semi-automated methods [16,17,18,19]. Considerable clinical and methodological heterogeneity can be observed, including number of patients, imaging modality, and the use of dichotomised configurations or pre-defined volume cut-off points; combined with the lack of a reliable gold standard to validate imaging parameters, this impacts the assessment of methodology accuracy and reproducibility, and therefore limits comparability across approaches.

In the clinical setting, there is a need to establish reliable methods that demonstrate high reproducibility and accuracy and are cost-effective in the acute setting, even if performed on imaging of different quality and density scale parameters and slice thickness, in order to determine the extent of infarcted tissue and amount of salvageable penumbra. The lack of international guidelines or consensus on the methodology for the determination of infarct volume in the acute phase greatly impacts the reporting of meaningful clinical data for the accurate interpretation and translation of effective therapeutic interventions. To this end, we carried out a systematic review to assess the current methodology for the reproducible and accurate estimation of infarct volume in patients suffering acute ischaemic stroke.

## 2. Methods & Design

Standardized reporting and registration: This systematic review was conducted in line with the Preferred Reporting Items for Systematic Reviews and Meta-Analyses (PRISMA) reporting guidelines (Supplementary Table S1) and was registered in PROSPERO, an international prospective register for systematic reviews (registration number CRD42024550583).

Search strategy and information sources: A comprehensive search of supporting evidence for current approaches used for assessing acute infarct volume from CT and MRI was conducted using Medline PubMed, Scopus, Cinahl, Cochrane Library, Web of Science, and Embase from the start of each database until May 2025, and screened using EndNote. MESH and keyword search terms were as follows: “Infarct volume assessment” [All Fields] OR “Infarct volume measurement” [All Fields] OR “Cerebral infarct volume” [All Fields] OR “Infarct volume determination” [All Fields] OR “Infarction volume assessment” [All Fields] OR “Infarction volume measurement” [All Fields] OR “Cerebral infarction volume” [All Fields] OR “Infarction volume determination” [All Fields]. Our search strategy was defined in consultation with a senior medical librarian. All searches were limited to human and English language.

Inclusion and exclusion criteria: The following inclusion and exclusion criteria were applied to search results: Inclusion criteria: (1) Image-based diagnosis of acute ischaemic stroke; (2) acute ischaemic stroke defined as <24 h; (3) human studies; (4) image-based diagnosis of acute ischaemic stroke by CT or MRI. Exclusion criteria: (1) animal model studies; (2) non-acute stroke studies; (3) article not available in English; (4) diagnosis of acute ischaemic stroke not confirmed by CT or MRI; (5) study does not present original data (letters; replies; commentaries; opinion articles).

**Figure 1:**
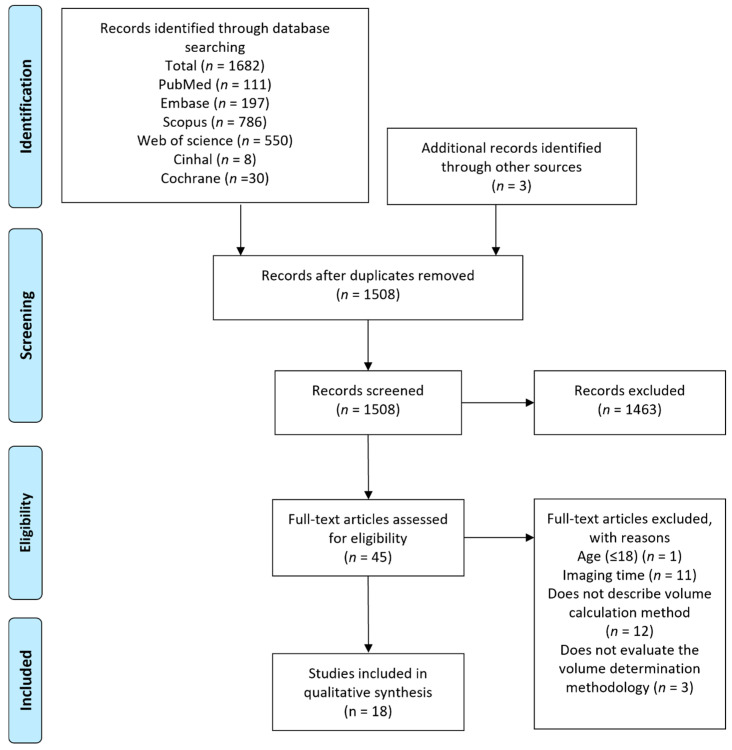
Flow chart detailing the study screening and selection process.

Study selection, data extraction, and qualitative synthesis: Following removal of duplicates, two reviewers independently applied the inclusion and exclusion criteria to titles and abstracts of all unique articles to assess for eligibility. Complete full-text articles were assessed for eligibility following title and abstract screening for final inclusion, with arbitration by a third reviewer in the event of no consensus. Reference lists of identified records were manually checked to identify additional relevant studies (Figure 1). For each study, two reviewers independently extracted and compared data on patient cohorts, imaging type and time from symptom onset, methodologies and quantification strategies, rater reliability, accuracy, sensitivity and specificity. Primary outcomes of interest included methodological accuracy and reproducibility (intra-rater, inter-rater and correlation) with secondary outcomes including sensitivity, specificity, agreement and predictive value. Data were synthesised in narrative and tabular formats. Data extracted from the included studies were classified and evaluated for the performance of techniques employed in the determination of acute infarct volume and are summarized in Tables 1 and 2. Characteristics of the included studies and information on inter-rater, intra-rater and methodological correlation, agreement, accuracy, sensitivity, specificity, and predictive value were tabulated.

Included studies quality and bias assessment: Included studies were assessed for risk of bias and applicability judgments by two reviewers in consultation with a biostatistician using the Quality Assessment of Diagnostic Accuracy Studies (QUADAS-2) tool [20]. QUADAS-2 items (patient selection, index test, reference standard, and flow and timing) were assessed in terms of risk of bias taking into consideration risk of systematic errors in the study design, conduct and reporting, and concerns regarding applicability with regard to patient selection, the utilized reference standard, and the method used in the determination of infarct volume and rated categorically (low risk, unclear, high risk).

## 3. Results

Included studies: The initial comprehensive search yielded 1685 articles eligible for screening. After removal of duplicates (177), 1508 articles were assessed for eligibility by title and abstract and 1463 articles were further excluded. Complete full-text analysis was carried out on 45 articles, and finally eighteen articles were included in the qualitative synthesis (Figure 1). Eighteen acute ischaemic stroke cohorts with a total of 3298 ischaemic stroke subjects were assessed; fifteen studies used DWI, two assessed NCCT, and one used NCCT and DWI. All studies included patients suffering ischaemic stroke in the acute phase, typically defined as onset ≤ 24 h, of which eight studies had populations within the 3–12 h window (<3, <6, <9, <12 h) [16,21,22,23]. Imaging time ranged from symptom onset of <6 to <24 h, and sample populations ranged from 30 to 1031 patients (Table 1). Across the included studies, a variety of manual, semi-automated, and automated approaches were compared (Table 1). Manual methodologies were defined as observer-dependent delineation of the region of interest, while semi-automated demarcation represents a combination of image analysis tools with rater judgment and refinement. Automated tools utilise specialised software to measure lesion volume in an automated fashion.

**Table 1:** Characteristics of the included studies.

Reference	Sample Size	Imaging Time (h)	Blinded to Clinical Data	Imaging Type	Method
Ay et al. [24]	58	<12	No	DWI	Manual planimetric delineation with subsequent automated volume estimation
Cauley et al. [25]	30	<24	Yes	NCCT	Digital image analysis algorithms using image intensity inhomogeneity correction and intensity thresholding
Fiebach et al. [26]	108	<24	Yes	DWI	ABC/2; Manual planimetric delineation; Maximum orthogonal diameter values
Han et al. [27]	79	<6	Unclear	NCCT DWI	Manual planimetric delineation; Automated software
Khoury et al. [28]	32	>6	No	DWI	Visual estimation based on reference standard infarct volumes
Khoury et al. [23]	36	>6	Unclear	DWI	Automated; Semi-manual infarct delineation with automated volumetric measurement
Kim et al. [29]	1031	<24	Unclear	DWI	Visual reference map-based estimation; semi-automated volumetric measurement with manual artefact removal
Kufner et al. [30]	238	≤9	Unclear	DWI	Diameter-based volume estimation; Manual planimetric delineation
Luby et al. [31]	193	<3	Unclear	DWI	ABC/2; Manual planimetric delineation
Luby et al. [32]	171	<3	Yes	DWI	Semi-automated infarct delineation with automated volumetric measurement
Pedraza et al. [21]	86	<12	Yes	DWI	ABC/2; Manual planimetric delineation
Rana et al. [33]	15	Acute (undefined)	Yes	DWI	Manual planimetric delineation
Sananmuang et al. [22]	109	≤24	Yes	DWI	ABC/2, ABC*/2 and manual planimetric delineation
Sims et al. [16]	63	<9	Unclear	DWI	ABC/2 and computerized planimetry
Steffenhagen et al. [34]	80	<12	Yes	DWI	Semi-automated computer-assisted volumetric analysis
Wong et al. [35]	875	≤24	No	DWI	Automated using deep learning model for infarct segmentation
Cimflova et al. [36]	44	≤24	Yes	DWI	Manual segmentation with automated algorithm (STAPLE) used to generate a computational reference standard
Cimflova et al. [37]	50	≤24	Yes	NCCT	Manually delineation with automated algorithm (STAPLE) used to generate a computational reference standard

DWI, diffusion-weighted imaging; NCCT, non-contrast computed tomography.

Manual and semi-automated methodologies: Manual techniques used included the ABC/2 method, based on a modification of the ellipsoid volume equation [16,21,22,26], visual estimation [26,28,29], manual delineation [25,26], and transverse diameter measurement [30]. The visual estimation approach utilised by Khoury et al. provided raters with no time constraints and permitted modification of image window and width levels. RAPID, an automated neuroimaging software program, was used as a validated comparison group following its exclusive use in the DAWN and DEFUSE 3 trials [28,38,39]. Raters provided a visual estimation of total infarct volume based on four reference RAPID infarct volumes (<21, <31, <51 and <71 mL). Raters were permitted to consult RAPID reference volumes at all times during their visual estimation evaluations. Kim et al. investigated visual estimation of infarct volume with the use of specialised reference maps corresponding to twelve known infarct volumes (0.05–19 mL) compared to semi-automated software estimates with manual artefact removal [29]. Fiebach et al. compared ABC/2 and manual delineation (using MRIcro 1.40) volumetric measurements with a simplified lesion size estimation approach, assessing only the maximum orthogonal DWI lesion diameters, measured by two resident physicians with 3 years of stroke imaging experience, blinded to clinical status [26]. Kuffner et al. assessed lesion diameters ≥ 3 cm where visible infarct was present on ≥3 consecutive DWI slices for sensitivity and specificity in predicting lesion volume ≥ 15 mL as determined by manual planimetric delineation for patient selection [30]. Rana et al. calculated lesion volume, apparent diffusion coefficient (ADC) and ratio of ischaemic to contralateral control ADC on DWI sequences; raters independently adjusted contrast and greyscale settings to individually optimise visualisation of lesion and grey/white matter and assessed the impact of altering the lesion boundary tracing by one pixel [33].

Cimflova et al. manually delineated infarct volumes slice by slice on DWI [36] and NCCT [37] scans 7 days apart, using ITK-SNAP software by three independent readers with >2 years’ experience [37]. Ischaemic lesion volume was calculated by multiplying the number of infarct voxels manually contoured with the resolution of the corresponding images [37] or by individual voxel volume measurements [36]. An automated algorithm, Simultaneous Truth and Performance Level Estimation (STAPLE), was used to establish the reference standard from combined manual segmentations, to which manual segmentations were compared.

Two studies employed semi-automated techniques in the identification of lesion area. Steffenhagen et al. used custom computer-assisted software QUANTOMO for semi-automated ROI generation to assist in volumetric measurements, with rater-defined final infarct boundary and volume interpretation [34]. Luby et al. (2006) [32] also utilised a semi-automated technique for slice segmentation of infarct area with subsequent manual refinement or correction on final lesion borders. Volumetric measurement was then automatically produced from total infarct area and slice thickness [32].

Automated methodologies: Khoury et al. investigated and compared three automated tools (Olea Sphere v3.0sp14, Brain Analyze, and RAPID) with five non-automated methods, involving semi-manual infarct delineation with subsequent automated DWI volumetric measurement calculated using various software (Telemis v480, Mango, MRICron, Carestream, or Syngo.via VB10B) [23,38,39]. Non-automated methods were carried out by experienced neuroradiologists (>5 years), blinded to measurements from automated tools, to determine the impact of discrepancies across methods on thrombectomy decisions when applying DAWN trial imaging criteria. Cauley et al. investigated the application of image pre-processing—image intensity inhomogeneity correction (IIC)—for sensitive identification of the subtle regional hypodensity commonly observed in early infarct NCCT, where sharp boundary delineation is difficult. The study applied two publicly available algorithms, the FSL automated segmentation tool (FAST) and ITK, which does not perform tissue segmentation, to estimate image intensity inhomogeneity and generate a “difference map” from the input image. The novel application of digital image analysis algorithms using IIC and intensity thresholding to early NCCT images was evaluated using both simulated infarcts and clinical cases and compared to manual freehand estimation of infarct volume [25]. Wong et al. [35] developed a deep learning model, whereby acute infarct volumes were manually segmented by three independent experts using MRIcro, and radiology report findings were used as the gold standard. Included patients were split 80/20 for training/testing (700/175 patients). Evaluation metrics were reported using the Dice score (a statistic to evaluate image similarity), precision, and recall, and were compared with manual labels.

### 3.1. Accuracy and Reproducibility: Visual Estimation

Khoury et al. investigated the specificity and accuracy of visual estimation of DWI infarct volume based on four available RAPID reference standard infarct volumes (<21, <31, <51, <71 mL) when comparing volumes automatically generated by RAPID software [28]. Accuracy of visual assessment for each volume cut-off point was 83% (66–94), 87% (71–95), 82% (65–93), and 76% (58–89), respectively. Moderate inter-rater agreement was observed, without significant difference in the level of experience, across all cut-off points (Table 2), with perfect agreement across all raters of 44%, 37%, 44%, and 50%, respectively. Sensitivity was reported to increase with infarct volume (61% (32–83) <21 mL; 82% (54–94) <31 mL; 89% (65–98) <51 mL; 96% (77–100) <71 mL), with 16.7% correctly estimating an infarct volume < 21 mL and 44.4% correctly estimating volumes < 71 mL in all patients according to the reference standard. Specificity was observed to decrease as infarct volumes increased (93% (75–98) <21 mL; 90% (69–97) <31 mL; 74% (49–90) <51 mL; 47% (24–70) <71 mL). The application of DAWN criteria to raters’ visual estimates of total infarct volume instead of RAPID measurements led to 19% erroneous thrombectomy decisions.

Kim et al. investigated the efficacy of visual estimation of infarct using defined volume reference maps (0.5, 1, 2, 3, 5, 7, 9, 11, 13, 15, 17, and 19 mL) on each template slice ranging (on the z-axis) from −15 to 51 mm. Reference maps with a similar infarct size at the corresponding z-axis level were used to estimate infarct volume over all slices and compared to semi-automated volumetric measurements with manual artefact removal [29]. A high correlation between reference map-based estimations and image analyser-based measurements (0.977) was reported; Bland-Altman plots demonstrated substantial agreement, with 4.6% (6/130) of cases being outside the limits of agreement. Disagreement between the methods increased with larger infarcts; in infarct volumes > 50 mL, reference map-based estimation underestimated infarct volume by 40%. When infarct volumes were classified as <21, <31, and <51 mL, accuracy (95.40% <21 mL; 94.60% <31 mL; 96.2% <51 mL) and specificity (100% <21 mL; 100% <31 mL; 100% <51 mL) of map-based infarct volume estimation was found to be excellent, with high sensitivity (88.90% <21 mL; 85.10% <31 mL; 86.50% <51 mL) (Table 2). The dichotomisation of infarct volume as <70 and ≥70 mL also yielded high accuracy (95.4%), specificity (100%), and sensitivity (77.8%). Inter-rater reliability using the visual reference map-based method by experienced and inexperienced raters was good, with 7.7% (10/130) of measurements outside the limits of agreement and a mean difference of 0.8 mL.

Cimflova et al. [36] used manual segmentations to calculate infarct volume on DWI by multiplying the number of infarct voxels by individual voxel volume measurements that were manually contoured using image resolution in the software; an automated algorithm (STAPLE) was utilised to generate a computational reference standard. The mean difference between manually segmented lesions and the reference standard for lesions measuring > 15 mL ranged from 5.0 ± 9.2 to 22.1 ± 14.8 mL, with an inter-rater ICC of 0.995 and intra-rater ICC of 0.985–0.994. For lesions measuring < 15 mL, the mean difference (±SD) was 0.7 ± 0.7 to 3.9 ± 2.4 mL, with inter-rater and intra-rater ICCs of 0.95 and 0.89–0.97. The mean absolute volume difference reported was 2.8 ± 6.8–13.0 ± 14.0 mL. The mean Dice similarity coefficient (DSC) for DWI infarct volume segmentations was 80.6 ± 11.7%–88.6 ± 7.5%. A subsequent study [37] by the group on NCCT reported mean manually segmented infarct volumes of 88.2 ± 91.5–135.5 ± 119.9 mL, with a mean reference standard of 92.5 ± 100.9 mL. Inter-rater ICC (95% CI) was 0.83 (0.76–0.88) and intra-rater ICC was 0.85 (0.72–0.92)–0.95 (0.91–0.97). The mean DSC for infarct volume segmentations across the three readers was 65.5 ± 22.9–76.4 ± 17.1%, and overall mean DSC was 72.8 ± 23.0%.

### 3.2. Accuracy and Reproducibility: ABC/2 Method

The reproducibility (inter-rater reliability) and reliability (intra-rater reliability) of the ABC/2 method was investigated across four studies [16,21,22,31]. In a study by Pedraza et al., the ABC/2 method was found to consistently overestimate infarct volume when compared with the planimetric method, with a median false increase of 7.33 cm^3^. The reproducibility of both ABC/2 and planimetric methods was excellent, with intra-class correlations of 0.992 and 0.985, respectively. Furthermore, all raters identified the largest area of infarction in the same slice in 65.1% of cases. The intra-rater reliability for the ellipsoid ABC/2 method was excellent (Pearson correlation, r = 0.998) (Table 2) [21].

Sims et al. demonstrated superior accuracy of the ellipsoid ABC/2 formula over cylindrical, bicone, and spherical ABC/2 approaches in acute stroke DWI (<9 h from onset), with significantly more values ranking closest to the values measured by computerized planimetry (31/63) compared with other Euclidean models (4/63, 17/63 and 11/63, respectively). The ellipsoid model also demonstrated excellent inter-rater (*R* = 0.965) and intra-rater (*R* = 0.992) reliability. This model was found to underestimate planimetric volumes by 10% across the range of measurements (95% CI (underestimate–overestimate), 328–255%), demonstrating the greatest variability in infarct volumes < 10 cm^3^ [16].

Similarly, Sanamuang et al. reported that the ABC/2 and adjusted (ABC*/2) method consistently underestimated infarct volume compared with manual planimetric segmentation. The ABC/2 method was found to perform superiorly (mean ± SD, 23.56 ± 48.81; median [min–max], 4.25 [0.11–318.94] cm^3^), with the adjusted ABC*/2 method obtaining significantly smaller volumes (mean ± SD, 13.37 ± 28.3; median [min–max], 2.08 [0.06–170.10] cm^3^) when compared to manual planimetry (mean ± SD, 28.5 ± 58.64; median [min–max], 5.56 [0.27–335.49] cm^3^). This was, however, for a combination of subacute (>24 h) and acute patients (<24 h). A subgroup analysis carried out on 14 acute participants demonstrated a statistically significant difference (*p* < 0.001) between manual planimetric volume (median [min–max], 15.95 [0.92–175.7]) and non-adjusted ABC/2 (median [min–max], 13.87 [0.12–154.4]), with results consistent with underestimation of the ABC/2 method in the full cohort analysis. The intra-class correlation (ICC) for inter-rater reliability indicated excellent reproducibility between the two raters for both non-adjusted (0.997) and adjusted (0.996) across a mixed subacute and acute cohort [22].

Strong inter-rater reliability of the ABC/2 method was reported by Luby et al., with Bland–Altman analysis of values from two independent raters demonstrating that 95% of measurements were within two standard deviations of the mean difference (Spearman correlation coefficient, 0.89) [31]. Comparison of ABC/2 and planimetric measurements demonstrated that 93.4% of the measurements were within two standard deviations from the mean difference (Spearman correlation coefficient 0.84) from 193 patients, while linear regression analysis demonstrated a strong correlation between the two methods (R^2^, 0.752; slope [CI], 0.867 [0.83–0.99]).

### 3.3. Accuracy and Reproducibility: Predictive Infarct Volume Diameter Methods

Fiebach et al. investigated the efficacy of the simplification of lesion size estimation, multiplying maximal orthogonal DWI lesion diameters (od-value) when compared to visual estimation and the ABC/2 method [26]. A good correlation between od-values and volumetric measurements on DWI was reported (Spearman correlation, 0.951), with increased accuracy of the od-value at larger volumes (*n =* 108). Identification of lesion volumes with od-values demonstrated a sensitivity and specificity of 90% and 98% for infarcts > 100 mL and 93% and 89% for infarcts > 70 mL. Visual estimation by two raters demonstrated a sensitivity of 50% and 60%, with 100% specificity for detection of infarcts > 100 mL, and a sensitivity of 53% and 87%, with a specificity of 100%, 91% for detection of infarcts > 70 mL (Table 2). The ABC/2 method demonstrated a 100% sensitivity for the detection of infarcts > 70 and >100 mL, with a specificity of 66% and 83% compared to visual and od-value ratings [26].

Kuffner et al. assessed lesion diameters ≥ 3 cm, present on ≥3 consecutive slices for sensitivity and specificity in predicting an infarct volume of ≥15 mL, as determined by manual planimetry [30]. Sensitivity and specificity were determined for cut off diameters of 3 cm (96.8% and 33.3%), 3.5 cm (96.8% and 50.6%), and 4 cm (91.7% and 61.7%) (Table 2). Low specificity at 3 cm was attributed to an over-estimation of ~23% cases, with 54 false positives where the largest extension of the infarct was ≥3 cm with an infarct volume < 15 mL. Increasing the cut-off diameter to 3.5 cm increased the specificity, correctly stratifying cases by < or ≥15 mL volume infarction, reducing overestimation of infarct volume to 17% cases. While a 4 cm cut-off increased specificity further, this was at the expense of sensitivity.

Rana et al. reported the apparent diffusion coefficient (ADC) and ratio of ischaemic to contralateral control ADC (ADCr) to demonstrate a greater reliability and reproducibility than manual volume measurement [33]. They reported manual volume measurement reliability (inter-rater (coefficient of variation (CoV) 85 ± 130%); intra-rater (CoV 20 ± 80%)) as significantly inferior to ADC (inter-rater (CoV 7.7 ± 19%), intra-rater (CoV 0.2 ± 12%)) and ADCr (inter-rater (CoV 8 ± 27%), intra-rater (CoV 0.8 ± 73%)) measurements (Table 2). Furthermore, alteration of the lesion boundary ROI by one pixel resulted in a systematic reduction in measured volumes of 22 ± 25% but altered ADC values by only 2.9 ± 4.9% and ADCr values by 2.7 ± 4.8%.

### 3.4. Accuracy and Reproducibility: Semi-Automated Methods

Two studies investigated solely inter-rater reliability [24,27] and two studies investigated both the intra-rater and inter-rater reliability of semi-automated methods [32,34]. Ay et al. assessed the inter-rater reliability of DWI manual delineation and subsequent automated computerised volume estimation based on slice thickness and manually defined ROI. The inter-class correlation between raters was excellent (*R* = 0.99); however, there was a negative correlation (Spearman correlation coefficient, −0.4), between lesion volume and individual measurement variability between examiners. Assessment of inter-rater agreement found that measurement error increased significantly to 20% with smaller infarct volumes (<5 mL), highlighting inter-rater measurement error with smaller lesions [24]. Similarly, Han et al. reported excellent inter-rater agreement (ICC [95% CI], 0.973 [0.958–0.983]) using a semi-quantitative DWI volume measurement of a manually outlined hyperintense area with semiautomatic thresholding, multiplied by slice thickness (Table 2) [27]. However, semi-quantitatively calculated infarct volumes were larger than infarct core volumes quantitatively calculated from ADC maps using commercial automated software. Steffenhagen et al. assessed patients presenting with transient ischaemic attack or minor stroke (NIHSS ≤ 3) within 12 h of symptom onset using the custom computer-assisted software QUANTOMO for semi-automated ROI generation and rater-defined final infarct boundary and volume interpretation [34]. Excellent inter-rater (ICC [lower 95% CI], 0.94 [0.88]) and intra-rater (ICC [lower 95% CI], 0.96 [0.86]) reliability was reported from acute DWI. Of note, 87% of participants had lesions < 5 mL (mean ± SD, 3.4 ± 7.4 mL) and minimum differences reported for inter-rater and intra-rater agreement were 4.9 and 4.0 mL, respectively.

Luby et al. reported good inter- and intra-rater reliability using semi-automated slice segmentation of the infarct area with subsequent manual refinement of final lesion borders and automatic volume measurement from the total infarct area and slice thickness [32]. Excellent intra-rater correlation was observed at acute (Spearman Coefficient, 0.965), 3 h (Spearman Coefficient, 0.961), and 24 h (Spearman Coefficient, 0.954) time points. Overall, intra-rater percent difference was 2–5% and inter-rater percent difference was <5% in acute phase measurements, with measurement error attributed to smaller infarcts (<10 mL).

### 3.5. RAPID vs. Automated and Non-Automated Methods

Khoury et al. investigated a number of automated (Olea Sphere v3.0sp14 [Olea Medical, France], Brain Analyze [BrainAnalyze, Versailles, France], and RAPID [IschemaView, Menlo Park, CA, USA]) and non-automated tools [23]. For non-automated methodologies, raters semi-manually delineated the ROI and infarct volume was automatically calculated (Telemis v4.80 [Telemis SA, Ottignies-Louvain-la-Neuve, Belgium], Mango [University of Austin, TX, USA], MRICron [University of South Carolina, Columbia, SC, USA], Carestream [Carestream Health, Rochester, NY, USA], and Syngo.viaVB10B [Siemens Healtheeners Global, Forchheim, Germany]). There was excellent correlation of global DWI infarct volume measurement across methods (ICC [95% CI], 0.89 [0.84–0.94]). However, after dichotomisation, while correlations were substantial for the pre-defined cut-off points (Fleiss’ kappa, κ > 0.6 for infarct volumes < 21 vs. ≥ 21 mL; κ > 0.7 for infarct volumes < 31 vs. ≥31 mL and <51 vs. ≥51 mL), disagreement occurred between at least one method in 2–42% of cases (Table 2). Automated methods yielded a higher number of cases with 100% agreement at all three cut off points (77.8–83.3%) compared to non-automated methods (69.4–80.6%) (Table 2). When applying the DAWN criteria, discrepancies between at least one method led to contradictory thrombectomy decisions in 33% of cases. Compared with RAPID, other methods lead to a contradictory thrombectomy decision (denying treatment to eligible patient or providing treatment to ineligible patient) in 6–9% (2/36–7/36) of cases, using the DAWN criteria. The number of cases with 100% agreement in thrombectomy decisions based on DAWN criteria was higher in automated methodologies (83.3%) compared to non-automated methodologies (77.8%) (Table 2) [23].

Employing a deep learning model to segment acute ischaemic stroke lesions, Wong et al. [35] reported high correlation in infarct volumes calculated from manual and automatic segmentation labels (0.999). When applied in a multivariate model using stroke volumes in 30 refined brain regions based upon modified Rankin Scale-relevance areas, and adjusted for clinical variables, the 90-day modified Rankin Scale outcome prediction AUC was 0.80 (95% CI, 0.76–0.83) with an accuracy of 0.75 (0.72–0.78).

### 3.6. Accuracy and Reproducibility: Automated Methods

Cauley et al. investigated infarct volume measurement using an automated pre-processing image intensity inhomogeneity correction (IIC) technique with different IIC algorithms (FSL and ITK) compared to manual freehand estimation. There was a strong correlation between the IIC FSL-algorithm infarct volume and manual volume measurements (Pearson’s r, 0.823). Manual measurements were only achievable in 14/41 cases with more mature infarcts due to the difficulty in identification of regional hypodensity in early infarct NCCT. Comparison of the performance of both IIC algorithms using simulated infarcts demonstrated an excellent correlation with true volume (Pearson’s r, 0.998). While both IIC algorithms were able to measure subtle hypodensities of acute infarcts and were highly correlated with each other, infarct volume measurements using ITK, which does not perform tissue segmentation, were found to be consistently smaller than those calculated using FSL. FSL segmentation algorithm was more robustly found to correlate with admission NIHSS, ASPECTS, and treatment decisions (tissue plasminogen activator) (Table 2) [25].

### 3.7. Included Studies Bias Assessment

QUADAS-2 assessment of the quality of included studies was high overall. Two studies were assessed as high risk of bias in patient selection for selecting cases for infarct volumes within defined volume cut-off points. Three studies were judged to be high risk of bias for index test and two at high risk of bias for reference standard, due to inadequate or lack of reference standard. Risk of bias was considered unclear for index test and reference standard where insufficient information was provided. One study scored high for bias in flow and timing for excluding poorly delineated hyperacute infarcts. Concerns for applicability were low for all included studies (Supplementary Figure S1).

## 4. Summary of Findings

### 4.1. Correlation

Out of 18 included studies, only 4 reported numerical correlation coefficients evaluating the agreement between infarct volume estimation methods and reference standards. Reported correlation values ranged from 0.238 to 0.999, reflecting varying degrees of agreement depending on the technique and comparator.

- High correlation values were found between volumetric measures and OD-value (r = 0.951), as well as in deep learning models (r = 0.999) [26,35]
- Intensity Inhomogeneity Correction (IIC) techniques showed correlations up to r = 0.998 when comparing measured and true lesion volume in synthetic infarcts [25].
- Lower correlation values (for example, r = 0.238) were observed when linking infarct volumes with clinical scales such as NIHSS and ASPECTS [25].

### 4.2. Reproducibility (Inter-Rater Reliability) and Reliability (Intra-Rater Reliability)

Thirteen of the included studies assessed the reproducibility (inter-rater reliability) and reliability (intra-rater reliability) of infarct volume estimation methods.

- Inter-rater and intra-rater reliability of the ABC/2 method was excellent, with reported inter-rater correlation values ranging from 0.89 to 0.997 and intra-rater correlation values ranging from 0.992 to 0.998 [16,21,22,31].
- Manual segmentation showed high inter-rater (0.997) and intra-rater (0.99) volumetric agreement on DWI; this was lower on NCCT, with inter-rater agreement as low as 0.27 on infarcts < 56.6 mL compared to 0.95 for infarcts < 15 mL on DWI [36,37].
- Semi-automated and automated methods reported low inter-rater correlations between visual methods when compared to RAPID (0.45–0.59). Excellent inter-rater correlation was observed for DWI infarct volume measurement between automated tools (0.91), semi-automated (0.90) tools, and across all semi-automated and automated tools (0.89). Moderate correlation values (0.77) were observed when applying automated and semi-automated infarct volumes to thrombectomy decisions based on the DAWN criteria [23,28].
- Semi-automated approaches with user refinement showed excellent inter-rater (0.973) and intra-rater correlation, including at acute (0.965), 3 h (0.961), and 24 h (0.954) time points. Lower inter-rater correlation values were observed when linking infarct volumes with clinical scales such as ASPECTS on NCCT (for example, 0.694), compared to DWI (0.94) [27,32].

**Table 2:** Performance of techniques employed by the included studies in the determination of acute infarct volume.

Reference	Technique	Correlation	Intra-Rater	Inter-Rater	Agreement	Accuracy	Sensitivity	Specificity	PPV	NPV
Sims et al. [16]	ABC/2		0.992 ^†^	0.965 ^†^						
Sananmuang et al. [22]	ABC/2			0.997 ^†^						
	Adjusted ABC*/2			0.996 ^†^						
Khoury et al. [28]	Visual assessment vs. RAPID									
	<21 mL			0.46 (0.31–0.64) ¶		83% (66–94)	61% (32–83)	93% (75–98)		
	<31 mL			0.59 (0.46–0.73) ¶		87% (71–95)	82% (54–94)	90% (69–97)		
	<51 mL			0.59 (0.46–0.71) ¶		82% (65–93)	89% (65–98)	74% (49–90)		
	<71 mL			0.45 (0.26–0.63) ¶		76% (58–89)	96% (77–100)	47% (24–70)		
Fiebach et al. [26]	Maximum orthogonal diameter (OD) vs. volumetric and ABC/2:									
	Volumetric vs. OD-value	0.951 ^††^								
	Observer 1 > 100 mL					90%	50%	100%	100%	89%
	Observer 2 > 100 mL					92%	60%	100%	100%	91%
	ABC/2 > 100 mL					76%	100%	66%	56%	100%
	OD-value > 100 mL					96%	90%	98%	90%	98%
	Observer 1 > 70 mL					86%	53%	100%	100%	89%
	Observer 2 > 70 mL					90%	87%	91%	81%	94%
	ABC/2 >70 mL					86%	100%	83%	59%	100%
	OD-value >70 mL					90%	93%	89%	78%	97%
Kufner et al. [30]	Lesion diameter vs. manual planimetry:									
	Identification of infarcts ≥ 15 mL: 3 cm cut-off						96.80%	33%		
	Identification of infarcts ≥ 15 mL: 3.5 cm cut-off						96.80%	50.60%		
	Identification of infarcts ≥ 15 mL: 4 cm cut-off						91.70%	61.70%		
Khoury et al. [23]	Automated vs. semi-automated tools									
	All tool measurements			0.89 [0.84–0.94] ^†^						
	Non-automated			0.90 [0.84–0.94] ^†^						
	Automated			0.91 [0.82–0.95] ^†^						
	All tool measurements < 21 versus ≥21 mL			0.62 [0.55–0.68] ¶	58.30%					
	Non-automated < 21 versus ≥21 mL			0.64 [0.53–0.74] ¶	69.40%					
	Automated < 21 versus ≥21 mL			0.64 [0.46–0.83] ¶	77.80%					
	All tool measurements < 31 versus ≥31 mL			0.70 [0.64–0.77] ¶	63.90%					
	Non-automated < 31 versus ≥31 mL			0.76 [0.66–0.87] ¶	75.00%					
	Automated < 31 versus ≥31 mL			0.70 [0.51–0.89] ¶	77.80%					
	All tool measurements < 51 versus ≥51 mL			0.74 [0.68–0.80] ¶	72.20%					
	Non-automated < 51 versus ≥51 mL			0.79 [0.68–0.89] ¶	80.60%					
	Automated < 51 versus ≥51 mL			0.77 [0.58–0.96] ¶	83.30%					
	Thrombectomy decisions based on DAWN criteria									
	All tool measurements			0.73 [0.67–0.79] ¶	66.70%					
	Non-automated			0.77 [0.67–0.88] ¶	77.80%					
	Automated			0.77 [0.58–0.96] ¶	83.30%					
Cauley et al. [25]	Inhomogeneity correction (IIC)									
	Measured vs. true lesion volume [synthetic infarcts]	0.998 ^‡^								
	Manual volume vs. IIC-FSL	0.823 ^‡^								
	IIC-FSL vs. IIC-ITK	0.766 ^‡^								
	IIC-FSL infarct volume correlation with admission NIHSS	0.544 ^‡^								
	IIC-ITK infarct volume correlation with admission NIHSS	0.238 ^‡^								
	IIC-FSL infarct volume correlation with ASPECTS	0.68 ^‡^								
	IIC-ITK infarct volume correlation with ASPECTS	0.68 ^‡^								
Rana et al. [33]	Manual		20 ± 80% ^§^	85 ± 130% ^§^						
	ADC		0.21 ± 12% ^§^	7.7 ± 19% ^§^						
	ADCr		0.8 ± 73% ^§^	8 ± 27% ^§^						
Luby et al. [32]	Semi-automated									
	DWI acute		0.965 ^††^							
	DWI 3 h		0.961 ^††^							
	DWI 24 h		0.954 ^††^							
Pedraza et al. [21]	ABC/2		0.998 ^‡^	0.992 ^†^						
	Manual Planimetric			0.985 ^†^						
Kim et al. [29]	Reference map-based									
	<21 mL					95.40%	88.90%	100%		
	<31 mL					94.60%	85.10%	100%		
	<51 mL					96.2%	86.50%	100%		
	Dichotomisation < 70 vs. ≥70 mL					95.4%	77.80%	100%		
Ay et al. [24]	Semi-automated software [DWI]			0.99 ^†^						
Han et al. [27]	Semi-automated software									
	NCCT ASPECTS			0.694 [0.523–0.804] ^†^						
	DWI ASPECTS			0.940 [0.90 –0.961] ^†^						
	DWI stroke volume			0.973 [0.95 –0.983] ^†^						
Steffenhagen et al. [34]	Semi-automated software [DWI]		0.96, 0.86 **	0.94, 0.88 **						
Luby et al. [31]	ABC/2 vs. planimetry	0.846 ^††^								
	ABC/2			0.89 ^††^						
Cimflova et al. [36]	Manual segmentation with consensus reference standard (generated with STAPLES algorithm)									
	Absolute infarct volume		0.991 (0.983–0.995) to 0.996	0.997 (0.995–0.998) ^†^						
	>15 mL		0.985 (0.965–0.994) to 0.994 (0.985–0.997) ^†^	0.985 (0.965–0.994) to 0.994 (0.985–0.997) ^†^						
	<15 mL		0.89 (0.75–0.96) to 0.97 (0.94–0.99)	0.95 (0.92–0.98) ^†^						
Cimflova et al. [37]	Manually delineation with consensus reference standard (generated with STAPLES algorithm)									
	Absolute infarct volume		0.85 (0.72–0.92) to 0.95 (0.91–0.97)	0.82 (0.76–0.88) ^†^						
	>56.6 mL		0.70 (0.42–0.86) to 0.94 (0.88–0.97) ^†^	0.78 (0.65–0.88) ^†^						
	<56.6 mL		0.52 (0.29–0.73) to 0.89 (0.77–0.95) ^†^	0.27 (0.14–0.46)^†^						
Wong et al. [35]	Deep learning model	0.999								
	Baseline multivariate model of clinical factors				0.72					
	Refined multivariate model of clinical factors				0.75					

^†^ Intraclass Correlation Coefficient [ICC, 95% CI]; ^‡^ Pearson Correlation Coefficient; ^§^ CoV, coefficient of variation ±SD; ^¶^ Fleiss’ Kappa (k), [95% CI]; ** Intraclass Correlation Coefficient [ICC, lower 95% CI]; ^††^ Spearman Correlation Coefficient; PPV, positive predictive value; NPV, negative predictive value; IIC-FSL, intensity inhomogeneity correction-FSL automated segmentation algorithm; IIC-ITK, intensity inhomogeneity correction-ITK algorithm; ADC, apparent diffusion coefficient; ADCr, ratio of ischaemic to contralateral ADC.

## 5. Discussion

The routine use and accessibility of NCCT and DWI make these imaging modalities the most representative imaging techniques used in the acute setting, informing decisions on approaches for reperfusion with thrombolytic therapy and endovascular thrombectomy. NCCT remains the modality of choice in the acute setting due to its speed, cost, and reduced exclusion criteria; however, early infarcts may show poor margin delineation. While MRI may facilitate detection of smaller infarcts at an earlier stage, MRI may be limited by availability and access; one study excluded 25% of patients due to incompatibility of imaging data with the local reading software [30]. Imaging time, quality, and interpretation warrant consideration in the choice and assessment of methodology for infarct volume assessment, especially in the setting of multi-centre trials. Minimising sources of variance may allow for more effective studies of stroke therapies and accelerate ischaemic stroke treatment decisions [24,25,27].

While the ASPECTS score is a widely validated powerful prognostic indicator of functional outcome and is widely applied, particularly to NCCT studies, several limitations of this scoring system exist [4,7,10,11,12,13,14,15]. ASPECTS is limited to the anterior circulation; territorial assessment is unequally weighted, and individual components of the ASPECTS score cover a variable amount of brain tissue [40]. The extent of early change to label a region affected is not clearly defined. Furthermore, conflicting evidence exists as to the utility of ASPECTS as a predictor of stroke outcome [40]. Correlation of estimated lesion volume between DWI-MRI and ASPECTS has been shown to vary considerably [41]. Reader interpretation may also vary depending on the experience of the reader and may be subject to bias.

The DAWN and DEFUSE 3 trials supported extended time windows for endovascular treatment. DAWN demonstrated the benefit of thrombectomy in unknown-onset and late-onset (>6 h) stroke patients who were found to have a mismatch between the severity of clinical deficit and infarct volume. Similarly, the EXTEND-IA trial demonstrated the benefit of early thrombectomy after the initiation of intravenous alteplase based on imaging findings in patients with a proximal cerebral arterial occlusion and salvageable tissue on CT perfusion imaging [42]. Infarct volume in these trials was assessed via DWI-MRI or CT perfusion via RAPID software. Although the utility of these studies is without question, timely access to imaging techniques including MRI and CT perfusion may be limited in some centres; widespread use of perfusion imaging is impacted by the cost–benefit ratio, including access, expertise, and time. Alternative methods of infarct volume assessment therefore are advantageous in order to identify patients with a mismatch between clinical severity and infarct volume.

Manual planimetry is subject to rater bias and is time consuming, limiting its application in acute clinical settings and therefore prompting interest in predictive diameter approaches [43]. Diameter-based manual planimetric delineation reported high sensitivity and low specificity secondary to overestimation of infarct volume; larger infarcts did not change the sensitivity, yet increased the specificity and reduced overestimation. Lesion site was not found to influence the estimation of lesion size. Diameter-based manual planimetric delineation is a robust method; however, in the clinical setting, overestimation should be considered [30]. Overestimation may result in clinical consequences; for example, if DAWN criteria are applied, discrepancies in volume estimation may result in patients transferring across treatment categories, which potentially excludes patients from treatment. Similarly, the employment of maximal orthogonal diameter values represents a simple alternative method to visual estimation; its excellent sensitivity and specificity for large volume infarcts may be useful for guiding therapeutic decisions [26]. Lesion volumetric assessment using ADC measurement and ratio of ischaemic–contralateral control ADC also demonstrates greater reliability and reproducibility than manual volume measurement [33]. In smaller lesions, there was greater variability in ADC measurement, which could be due to the partial volume effect. Clinically, ADCr changes over time; however, this does not imply recovery but the evolution of tissue damage [33].

ABC/2 is a reproducible technique with very high observer reliability, with the exception of with larger infarct volumes, where it demonstrates low accuracy. In the acute clinical setting, ABC/2 is a simple, rapid technique that can be performed in real time and may be useful for infarct volume estimation and informing treatment decisions, as long as the limitations are recognised. Given that these studies assessed the performance of this approach on MR imaging, it is important to note that the use of the ABC/2 method on CT images in the hyper-acute setting will likely demonstrate poorer reliability, given the difficulty of identifying infarct margins <3 h [16,21,22,26,31].

All studies employing semi-automated software approaches found the accuracy of the software to be inversely correlated with infarct size; intra-rater and inter-rater measurements were close, with inaccuracies in patients with small lesions [23,24,32,34]. Semi-automated methods are reliable, rapid approaches that require short learner input; however, the reproducibility of the software can be affected by technical variables such as image quality, processing, and software analysis. Further challenges when using semi-automated software are presented with the requirement of experienced readers for the determination of the infarct boundary [29,32]. Fully-automated systems can automatically outline lesion borders; however, they are expensive, not available across all medical centres, and the presence of another acute or chronic ischaemic lesion in the same slice may lead to miscalculation [24,25]. Commercially available image display and analysis programs were found to consistently produce increased measurement error as the lesion volume decreases, likely related to rater judgment, inclusion or exclusion of sulcal areas, encephalomalacia, or hematoma [34].

The assessment of a number of automated and semi-automated methodologies, where raters semi-manually delineated the ROI and infarct volume was automatically calculated, demonstrated high reliability across all methods. Discrepancies were observed between all tools when compared to RAPID as the reference standard, which is attributable to a number of factors, including rater involvement, differences in operating algorithm, or the lack of standardized DWI acquisition parameters that might alter the performance of some automated tools [23]. However, overall, all semi- and automated software offered similar performances. With this in mind, it is worth taking into consideration that there is an absence of good evidence regarding the reliability of RAPID automated imaging analysis, which is used as the reference standard for these tools. RAPID software is designed to be a decision support tool, not for use in isolation but to be read in conjunction with a radiologist’s review. Automated infarct volume measurement accuracy improves with increasing true infarct size and infarct attenuation, and sensitivity to small changes in radiodensity can help identify infarcts before becoming visible on the routine images [25].

Accurate assessment of infarct volume is a valuable tool in a patient-centred approach to clinical assessment and treatment, offering prognostic information and implications for the selection and success of acute therapeutic intervention such as thrombolysis and thrombectomy, which are currently time-dependent [10,11,12]. For many of the above volume assessment methodologies, it is clear that larger infarct volume and, to a lesser extent, small infarct volume impacted rater reliability, which in turn may impact treatment decisions. The DAWN trial provided evidence for an extended time window for thrombectomy beyond 6 h based on clinical imaging mismatch and relied solely on infarct volume for patient selection. Infarct volume cut-off points in the DAWN trial are set at small volumes; when applying DAWN criteria for decision making, the under- or overestimation of infarct volume may result in erroneous treatment decisions, potentially precluding eligible patients from treatment in such trials or erroneously assigning a particularly outcome to a patient with an infarct of a certain size. Ultimately, there is a need for accurate measurement of infarction from imaging, allowing for a better evaluation of the relationship between outcome and variable infarct size.

Manual assessment of infarct volume does not require access to specialised software but is impacted by level of experience and rater over- or underestimation of lesions impacts on accuracy and repeatability. The application of the ABC/2 method is highly reliable, reproducible, and accurate and provides the best manual estimate of infarction, with the caveat that it also has a tendency to under- or overestimate infarct volume, as this is still a form of estimation of actual volume. Any manual application of infarct volume estimation will be subject to degrees of rater bias, and to this end, semi-automated and automated computational approaches can attenuate rater-introduced variability; however, these are not without their own limitations. While semi-automated and automated tools to measure infarct volume offer similarly high rates of reliability, none report perfect agreement. Discrepancies with automated software can result from differences in operating algorithms and, importantly, lack of the standardisation of image acquisition parameters, quality, and format may impact performance and reproducibility, particularly in a multi-centre setting. Moreover, abnormalities frequently lack defined contrast boundaries and may overlap with non-pathological structures. While it is not possible to draw firm conclusions about the sensitivity and specificity of any one of these techniques, of all the methods assessed, automated and semi-automated approaches utilising rater judgment and refinement represent the most promising methodologies in volumetric determination.

## 6. Limitations

There are a number of limitations to this systematic review. In one study there was difficulty obtaining informed consent, which introduces a bias against very large infarctions as these patients often are unable to give informed consent [26]. Several studies included only a small number of patients, impacting the assessment of methodology accuracy and reproducibility. Only three studies assessed acute NCCT, which remains the modality of choice in most centres, and access to MR imaging techniques may be limited in some centres. Furthermore, some studies only assessed measurements in dichotomised configurations or pre-defined volume cut-off points, impacting the applicability outside trial selection and the evaluation of outcomes related to infarct volume. Important methodological details, including precise imaging parameters and whether imaging was read blinded, were not given in all studies to allow for an accurate comparison of the efficacy of any one method employed. Study reports can be variable, not every study assessed sensitivity and specificity, and not all image readers were blinded to patient clinical information. Also, results can vary based on the size of the lesion and human factor variability, such as individual performance, software settings, and biases. Finally, the lack of a reliable gold standard is lacking to validate imaging parameters, which therefore limits comparability across approaches. It is also worth acknowledging the limitations of current imaging techniques in their ability to accurately determine the extent of irreversibly injured tissue on baseline imaging, which will ultimately impact the severity of the infarcted tissue and outcome. Despite the ability to determine the core volume, uncertainty surrounding the viability of affected tissue, which may evolve to full or partial infarction or is potentially viable with early reperfusion, means that core infarct volume is not an adequate indicator of the ultimate burden of irreversibly damaged tissue [44]. Recently, a number of large infarct thrombectomy trials have suggested the possible benefits of EVT in some patients with large vessel occlusion (LVO) and large infarction cores [45,46].

## 7. Conclusions

Of all the methods, the automated and semi-automated approaches utilising rater judgment and refinement represent the most robust approaches, with semi-automated tools demonstrating consistent and repeatable results. The best results were observed using semi-automated tools on DWI, demonstrating excellent intra- and interrater concordance and consistent and repeatable results. Further studies should be designed and conducted to comprehensively assess the accuracy and reproducibility of these approaches and, importantly, their feasibility within the acute setting. Furthermore, we recommend the standardisation of reporting of study methodologies to increase reliability and reproducibility in the reporting of meaningful clinical data. Standardised uniform reporting will serve to enhance the transparency and reproducibility of stroke trials and methodology, especially in a multi-centre setting, and allow for the accurate interpretation and comparison of the efficacy of therapeutic interventions and patient outcomes. Minimising sources of variance may allow for more effective studies of stroke therapies and accelerate ischaemic stroke treatment decisions.

## Supplementary Materials

The following supporting information can be downloaded at: https://www.mdpi.com/article/10.3390/brainsci15060583/s1. Figure S1. Risk of bias assessment. Quality of included studies was assessed in terms of risk of bias and concerns regarding applicability. Bars represent proportion of studies with associated risk levels (low, unclear, high) with regard to patient selection, utilized reference standard, and technique employed (index test). Table S1. PRISMA 2020 Checklist. Ref. [47] is cited in the Supplementary Materials file.
