# Supplementary material for: Tissue Is the Issue: A Systematic Review of Methods for the Determination of Infarct Volume in Acute Ischaemic Stroke
Source: Brain Sci. 2025 May 28;15(6):583. doi: 10.3390/brainsci15060583 (PMC12190565; doi:10.3390/brainsci15060583)
Supplement: Supplementary file 1 [file brainsci-15-00583-s001.zip › AlAhmed 2025_Supplementary Figure S1.pdf]

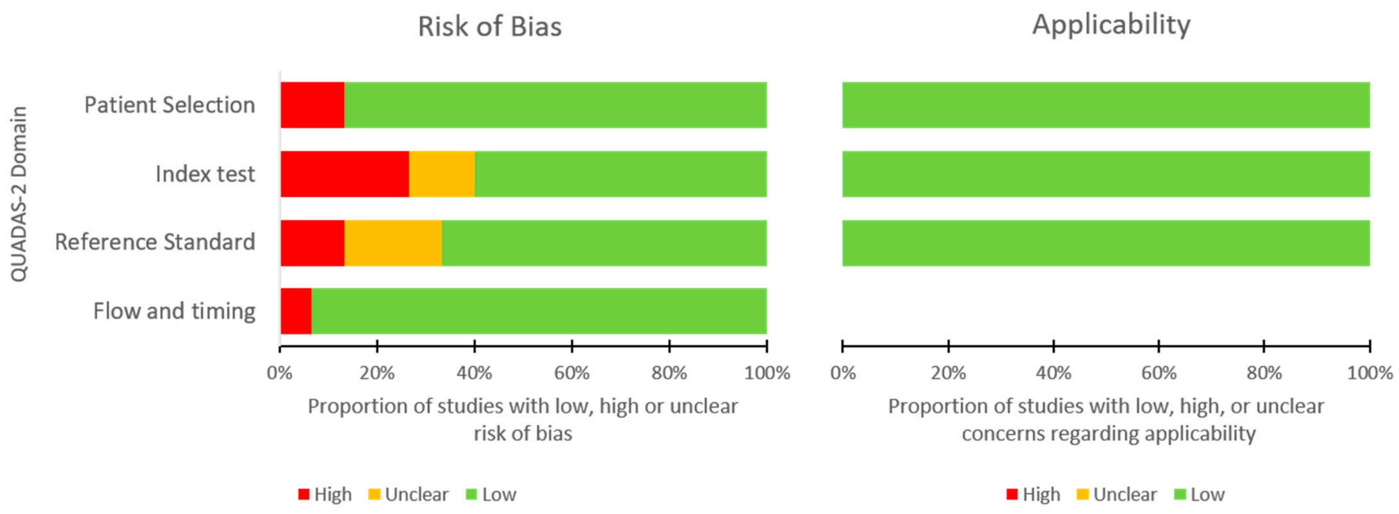

Supplementary Figure S1. Risk of bias assessment. Quality of included studies was assessed in terms of risk of bias and concerns regarding applicability. Bars represent proportion of studies with associated risk levels (low, unclear, high) with regard to patient selection, utilized reference standard, and technique employed (index test).
